# Genomic Investigation of Two *Acinetobacter baumannii* Outbreaks in a Veterinary Intensive Care Unit in The Netherlands

**DOI:** 10.3390/pathogens11020123

**Published:** 2022-01-20

**Authors:** Soe Yu Naing, Joost Hordijk, Birgitta Duim, Els M. Broens, Linda van der Graaf-van Bloois, John W. Rossen, Joris H. Robben, Masja Leendertse, Jaap A. Wagenaar, Aldert L. Zomer

**Affiliations:** 1 Department of Infectious Diseases and Immunology, Faculty of Veterinary Medicine, Utrecht University, 3584 CM Utrecht, The Netherlands; s.y.naing@uu.nl (S.Y.N.); joost.hordijk@rivm.nl (J.H.); b.duim@uu.nl (B.D.); e.m.broens@uu.nl (E.M.B.); l.vandergraaf@uu.nl (L.v.d.G.-v.B.); mleendertse@alrijne.nl (M.L.); j.wagenaar@uu.nl (J.A.W.); 2 Department of Medical Microbiology, University Medical Center, University of Groningen, 9700 AB Groningen, The Netherlands; j.w.a.rossen@rug.nl; 3 Department of Pathology, University of Utah School of Medicine, Salt Lake City, UT 84112, USA; 4 Department of Clinical Sciences of Companion Animals, Faculty of Veterinary Medicine, Utrecht University, 3584 CM Utrecht, The Netherlands; j.h.robben@uu.nl

**Keywords:** *Acinetobacter baumannii*, whole-genome sequencing, antimicrobial resistance, veterinary medicine

## Abstract

*Acinetobacter baumannii* is a nosocomial pathogen that frequently causes healthcare-acquired infections. The global spread of multidrug-resistant (MDR) strains with its ability to survive in the environment for extended periods imposes a pressing public health threat. Two MDR *A. baumannii* outbreaks occurred in 2012 and 2014 in a companion animal intensive care unit (caICU) in the Netherlands. Whole-genome sequencing (WGS) was performed on dog clinical isolates (*n* = 6), environmental isolates (*n* = 5), and human reference strains (*n* = 3) to investigate if the isolates of the two outbreaks were related. All clinical isolates shared identical resistance phenotypes displaying multidrug resistance. Multi-locus Sequence Typing (MLST) revealed that all clinical isolates belonged to sequence type ST2. The core genome MLST (cgMLST) results confirmed that the isolates of the two outbreaks were not related. Comparative genome analysis showed that the outbreak isolates contained different gene contents, including mobile genetic elements associated with antimicrobial resistance genes (ARGs). The time-measured phylogenetic reconstruction revealed that the outbreak isolates diverged approximately 30 years before 2014. Our study shows the importance of WGS analyses combined with molecular clock investigations to reduce transmission of MDR *A. baumannii* infections in companion animal clinics.

## 1. Introduction

*Acinetobacter baumannii* (*A. baumannii*) is an opportunistic pathogen commonly associated with nosocomial infections and poses a critical threat in healthcare settings. It can cause fatal infections such as bloodstream infections and pneumonia in humans and animals [1,2,3,4,5]. The emergence of multidrug resistance (MDR) of *A. baumannii* in nosocomial infections was reported for the first time in the early 1980s [6]. Antimicrobial resistance (AMR) in *A. baumannii* is evolving rapidly, leading to extensive drug resistance against available antimicrobials, including carbapenems and third-generation cephalosporins, the last resort drugs to treat serious bacterial infections [7,8]. In addition, *A. baumannii* has an 86-kb resistance island carrying 45 different genes associated with antimicrobial resistance [9], and its propensity to rapidly acquire resistance genes from other bacterial species and develop resistance during the middle of treatment may limit therapeutic options [10]. This threat prompted the World Health Organization (WHO) to prioritize the research and development pipelines to discover new antimicrobials for carbapenem-resistant *A. baumannii* in 2017 [11].

Despite extensive research on *A. baumannii* in human medicine, it remains a neglected pathogen in the veterinary and environmental health sectors [5,8,12]. Previous studies have suggested that *A. baumannii* might have an animal reservoir since *A. baumannii* has been isolated from different animals, including pets [2,3,13], food-producing animals [7,12], and living vectors such as lice [13]. Multiple reports have been published on *A. baumannii* in companion animals, including dogs, cats, and horses [7]. It has been demonstrated previously that *A. baumannii* can also survive on the skin of healthy dogs [14]. This commensal skin carriage may be a potential reservoir for veterinary nosocomial infections. Recently, a New Delhi Metallo-beta-lactamase 1 (NDM-1) positive, carbapenem-resistant *A. baumannii* strain was reported for the first time in a dog in Europe [15], and their findings suggested that companion animals may have accidentally acquired NDM-1 producing strains from humans. In addition, companion animals and humans can share the identical clones of *A. baumannii.* Still, data from animal origin remain too limited to understand the animal-human interplay of *A. baumannii* [5,16]. In human hospitals, infection prevention and control (IPC) measures are in place [17], but standardized IPC measures in the veterinary setting are limited, just as epidemiological surveillance programs [18]. Only a few studies have investigated the transmission chain and epidemiology of *A. baumannii* in veterinary clinics and hospitals [8,19,20]. Whereas the zoonotic potential of methicillin-resistant *Staphylococcus aureus* (MRSA) and extended-spectrum beta-lactamase (ESBL) carrying *Escherichia coli* are studied intensively, little attention is paid to exploring the potential of multidrug-resistant (MDR) *A. baumannii* as a zoonotic pathogen [19,21]. A few outbreaks of *A. baumannii* were described in veterinary clinics in Europe [3,5,13]. As a protracted outbreak example, a single clone of *A. baumannii* was present in different wards of a veterinary hospital in Germany from 2000 to 2008 [21].

Previously, the outbreaks of *A. baumannii* were studied using conventional molecular tools such as polymerase chain reaction (PCR) [22] or Pulsed Field Gel Electrophoresis (PFGE) [23]. However, traditional molecular typing methods often lack the resolution for strain differentiation in nosocomial settings [24]. In contrast to conventional typing approaches, whole-genome sequencing (WGS) ushered in a new era of outbreak management, with some excellent examples of how the increased resolution was beneficial in managing hospital outbreaks [25,26,27]. WGS, in combination with core-genome multi-locus sequence typing (cgMLST), provides the highest discriminatory power for outbreak investigations and an optimal resolution for studying the relatedness of outbreak strains.

This study describes two outbreaks of multidrug-resistant (MDR) *A. baumannii* in 2012 and 2014 in the companion animal intensive care unit (caICU) of Utrecht University in the Netherlands. The main aim of this study was to investigate the relatedness of two outbreaks using epidemiological data and genome sequences from animal and environmental isolates from the caICU. To our knowledge, this is the first genome-based outbreak investigation veterinary study comparing different typing methods, including the conventional MLST typing, core-genome MLST (cgMLST), pan-genome analysis, and single nucleotide polymorphisms (SNP)-based molecular clock analysis.

## 2. Results

### 2.1. Description of A. baumannii Outbreaks and Isolates Characteristics

Two outbreaks of *A. baumannii* occurred in 2012 and 2014 at the caICU of the Faculty of Veterinary Medicine, Utrecht, the Netherlands. The first outbreak took place from June to September 2012, and the isolates were recovered from four separate dogs at different time points in the caICU. Each patient was admitted to the ICU at different time points, and there was no overlap of ICU stay among the four dogs. An additional 25 environmental samples from the caICU were obtained. However, they were all negative for A. baumanii. The second outbreak took place in March 2014 in the caICU, on which two dogs were infected with A. baumanii. One patient (214030705701) was admitted and stayed in the ICU for complications following surgery, and another one (214031705301) was never admitted to the ICU but stayed in a medium care ward for recovery across from the ICU ward. In the 2014 outbreak, A. baumanii was recovered from 18 of 28 environmental screening samples, including the caICU treatment table, a cage, the operating table, the preparation room, and the fur of a hospitalized dog. The antimicrobial susceptibility tests (ASTs) revealed that all clinical isolates from both 2012 and 2014 outbreaks and one environmental sample from 2014 (214032504901) were multidrug-resistant (MDR), conferring resistance to aminoglycosides, cephalosporins, chloramphenicol, enrofloxacin, penicillins, tetracycline, and trimethoprim/sulfamethoxazole. The phenotypic resistance of other environmental isolates from 2014 were identical, showing resistance to third-generation cephalosporins (3GC), chloramphenicol, and penicillins. The MLST analysis using the Pasteur scheme revealed that all outbreak isolates and one surface isolate from the ICU treatment table belonged to the same sequence type (ST2). In contrast, other environmental isolates displayed ST241, ST239, and ST837. One isolate (214032504501) collected from a cage had an unknown or untypeable sequence type (ST). The description of bacterial isolates and genomic characterization are summarized in Table 1. AST results can be found in Supplementary Table S1, and the epidemiological features of the two outbreaks are visualized in Supplementary Figure S1.

### 2.2. Outbreak Investigation Using Core-Genome MLST (cgMLST)

**Figure 1:**
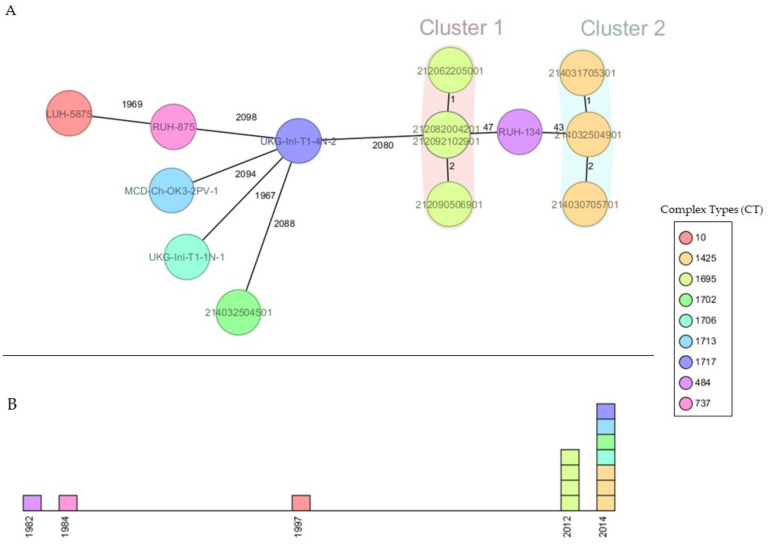
Outbreak investigation of MDR *A. baumannii* isolates in companion animal intensive care unit (caICU) in the Netherlands using cgMLST. (**A**) The minimum spanning tree of *A. baumannii* isolates is based on 2390 target genes of core genome MLST (cgMLST). The nodes are colored by complex types (CT) provided by cgMLST. Isolate IDs are labeled in the nodes, and the numbers between each circle indicate the cgMLST SNP differences between the isolates. The highlighted clonal clusters represent closely related genotypes (≤10 different alleles). (**B**) An epidemic curve of *A. baumannii* infections in which different colors correspond to different complex types (CT).

Based on the MLST finding of the same sequence type (ST2) with identical resistance patterns in outbreak isolates, it was assumed that the *A. baumannii* 2012 outbreak strains somehow thrived in the caICU for protracted times. To confirm the relatedness of the outbreak strains, the whole-genome sequences of outbreak isolates were compared with sequences of three human reference strains (Table 1), which are dominant in Europe. All genomes contained >93% of 2390 alleles defined in the cgMLST scheme. The 14 isolates were grouped into 9 distinct clusters based on cgMLST complex types (CT) (Figure 1). The cgMLST analysis identified two clonal clusters (C1 and C2) with different complex types (CT1695 and CT1425) in which only 1 or 2 alleles differences were found within each cluster. C1 consisted of four clinical isolates from the 2012 outbreak, whereas C2 was formed by two clinical isolates and one surface isolate derived from the caICU treatment table (214032504901) from the 2014 outbreak. Both clusters were closely related to RUH-134, the European Clone-II human reference strain, in which its alleles differed by 47 and 43 single nucleotide substitutions (SNPs) from C1 and C2, respectively. The environmental isolates belonged to distinct ST types separated by >2080 SNP differences from the C1 and C2 and reference strains (Figure 1).

### 2.3. Estimation of Divergence Date of ST2 Outbreak Isolates

A SNP-based molecular clock analysis was performed to estimate the mutation rate on an evolutionary time scale of the ST2 outbreak isolates. The maximum SNP difference between outbreak-related ST2 isolates was 421 SNPs, but most SNPs were obtained by recombination events that took place in the ST2 outbreak isolates, indicated by the fact that 75% (317/421) SNPs were located in regions < 1 kb apart (Supplementary Table S3). The exclusion of these recombination regions revealed that the 2012 and 2014 outbreak isolates differed by only 84 SNPs. A Bayesian molecular clock analysis allows indicating the time of divergence of the ST2 outbreak isolates (i.e., the mutation rate of the SNPs difference identified from the ST2 outbreak isolates). The molecular clock was estimated at 1.286 × 10^−6^ (95% highest posterior density (HPD) interval 1.125 × 10^−6^−1.449 × 10^−6^) substitutions per site per year. This estimated substitution rate referred to approximately five to seven SNPs per year. Based on this analysis, the divergence date of the 2012 and 2014 outbreak isolates was 30 years ago (95% HPD interval 25–35), suggesting that the ancestor of the 2012 and 2014 outbreak isolates may have appeared around the 1980s.

### 2.4. Comparative Genome Analysis

**Figure 2:**
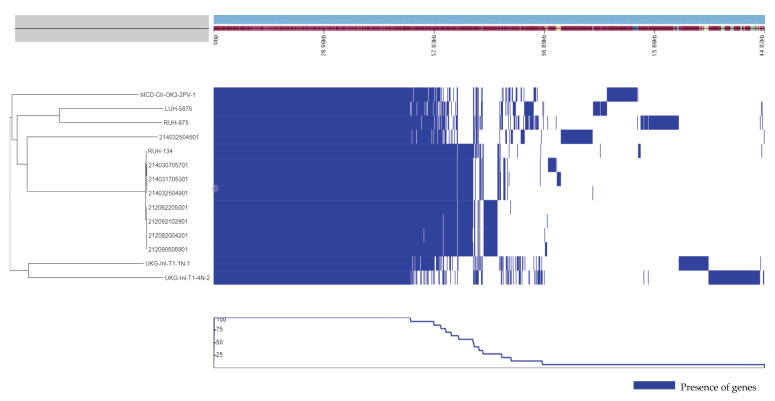
The differences in gene content between genomes included in this study. The pan-genomic matrix (right block) shows the absence and presence of core and accessory genes corresponding to mid-rooted phylogenetic dendrogram (left) (blue = presence of genes, white = absence of genes). The red line (top) indicates the size of contigs with different kilobase (kb). The blue line curve underneath the matrix displays the frequency of the presence of genes in each genome.

The phylogenetic analysis based on core genome alignment with SNP detection demonstrated that the outbreak isolates belonged to different cgMLST clusters and were genetically closely related to the EC-II human reference strain. Two environmental isolates from 2014 (one from the preparation room (UKG-Inl-T1-4N-2) and one from the clinic operating table (UKG-Inl-T1-1N-1) belonged to a distinct cluster from the rest of the isolates (Figure 2). The pangenome analysis showed that all genomes shared 2588 core genes with differences in gene presence and absence between outbreak isolates. The gene differences between the 2012 and 2014 outbreak isolates included several phage components, a potential capsular biosynthesis region, several genomic islands, and mobile genetic elements containing antimicrobial-resistance genes (not shown). Serum resistance gene (*traT*) was present only in a dog genome with wound infection from 2012 (212090506901). There were no host-associated genes identified in the human and animal isolates. However, we identified some virulent genes associated with ST2 isolates. Virulence genes such as biofilm-associated protein (*bap*) and TonB dependent siderophore receptor (*bauA*) were only found in ST2 isolates. The environmental isolates carried unique accessory genes that were not detected in clinical isolates. For example, genes coding for a type IV secretion system protein complex was detected only in one sample isolated from the clinic operating table (UKG-Inl-T1-1N-1). The variation of gene content differences between isolates is displayed in Figure 2.

### 2.5. Antimicrobial Resistance Genes (ARGs) and Mobile Genetic Elements (MGEs)

**Figure 3:**
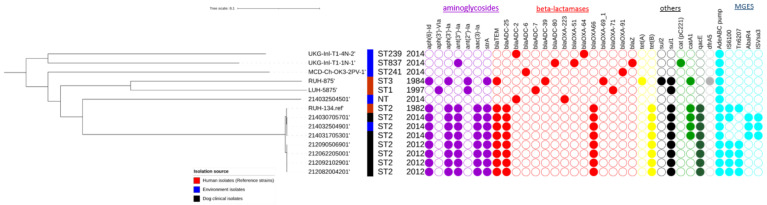
The presence and absence of antimicrobial resistance genes (ARGs) and mobile genetic elements (MGEs) associated with ARGs in genomes included in this study. The isolation source was represented by colored squares (red = human, blue = dog, black = environment). The colored circles indicated the presence of genes in which different colors showed different classes of ARGs (purple = aminoglycosides, red = beta-lactam, yellow = tetracyclines, black = sulfonamides, green = chloramphenicol), antiseptic (dark green = quaternary ammonium compound-resistant protein, *qacE*), *dfrA5* (light grey) and efflux pumps (AdeABC), and MGEs colored in cyan.

All ST2 *A. baumannii* isolates harbored the Acinetobacter derived AmpC ADC-25 cephalosporinase (*bla*ADC-25) in their chromosomes, conferring resistance to cephalosporins, while the non-ST2 isolates had different *bla*ADC variants (*bla*ADC-2, -6, -7, -39, -80) (Figure 3). The ST2 isolates shared identical beta-lactamase genes such as *bla*ADC-25, *bla*OXA-66, and *bla*TEM, where other (non-ST2) isolates displayed different *bla*OXA genes (*bla*OXA-223, -OXA-51, -OXA-64, -OXA-69, -OXA-71, and -OXA-91). None of the patient genomes carried any carbapenemase genes. The acquired carbapenemase Ambler Class D gene *bla*OXA-51 and the beta-lactamase gene *blaZ* were only present in a sample collected from the clinic operating table (UKG-Inl-T1-1N-1). All ST2 isolates furthermore carried the same ARGs conferring resistance to aminoglycosides (*aph(6)-Id*, *aph(3′)-Ia*, *ant(3″)-Ia*, *aac(3)-Ia*), tetracycline (*tet*(B)), and sulfamethoxazole (*sul1*). Antiseptic resistant genes (*qacE*) conferring resistance to chlorhexidine, benzylkonium chloride, ethidium bromide, cetylpyridinium chloride were found in ST2 isolates, but the *qacE* gene was not found in the environmental ST2 isolates. Although both the 2012 and 2014 outbreak isolates were phenotypically resistant to chloramphenicol (Supplementary Table S1), the chloramphenicol acetyltransferase (*catA1*) gene encoding for chloramphenicol resistance was only present in the 2014 outbreak isolates, but not in the 2012 isolates. The resistance-nodulation-division (RND) type AdeABC multidrug resistance efflux pump that enables to pump out aminoglycosides, trimethoprim, chloramphenicol, fluoroquinolones, tetracyclines, and ethidium bromide was present in all isolates.

The isolates of the two outbreaks carried different mobile elements carrying resistance genes, and different mobile genetic elements such as insertion sequences (IS) and unit transposons were identified. The 2012 clinical isolates had IS6100 insertion sequence carrying AMR genes *aadA1*, *aac(3)-Ia*, *sul1* and *qacE*, and Tn_6207_ transposon carrying tetracycline- and streptomycin-resistant genes (*tet*(B) and *aph(6)-Id*). The 2014 clinical isolates had a different IS type (ISVsa3) and AbaR4 transposon carrying tetracycline- and streptomycin-resistant genes (*tet*(B) and *aph(6)-Id*).

With RFPlasmid and Plasmidfinder, contigs containing replication (*rep*) genes were detected in two environmental samples (UKG-Inl-T1-1N-1, UKG-Inl-T1-4N-2), but not in the clinical isolates, indicating that only the environmental samples contain putative plasmids. One environmental sample (UKG-Inl-T1-1N-1) collected in 2014 from the operating room of the clinic carried a putative plasmid containing both a *rep*_7a_ gene and chloramphenicol resistant gene (*cat*_pC221_). The other environmental isolate (UKG-Inl-T1-4N-2), collected from the preparation room, carried a putative plasmid containing a replication and *tet*(R) gene. Other environmental isolates had only beta-lactamase genes and an efflux pump (AdeABC) and did not carry any mobile genetic elements associated with resistance mechanisms. The antimicrobial genes and relevant MGEs are displayed in Figure 3.

## 3. Discussion

The current study findings captured the genomic epidemiology of two MDR *A. baumannii* outbreaks in 2012 and 2014 at the caICU in the Netherlands. The canine isolates from both outbreaks shared the same MLST sequence type (ST2) and identical phenotypic resistance pattern suggesting a protracted outbreak. The canine outbreak isolates are genetically similar to the European clone (EC-II), one of the most prevalent clones globally. Even though this study could not identify how MDR *A. baumannii* strains were introduced to the veterinary clinic, we proved that MDR clones were shared among humans, companion animals, and the environment. One environmental sample from 2014 (214032504901) shared the same genotype as ST2 clinical isolates, but other samples from the ICU environment were genetically distinct from clinical and reference strains. The WGS analysis also revealed that the MDR-*A. baumannii* isolates from both outbreaks in the caICU diverged 30 years before 2014, consistent with the spread of MDR *A. baumannii* in the early 1980s [29].

### 3.1. Two Independent MDR A. baumannii Outbreaks Confirmed by WGS-Based Analysis

The two outbreaks might have been mistaken as a single protracted one if the interpretation was solely based on the same MLST sequence type (ST2) and phenotypic resistance profiles. Indeed, two different clonal clusters of the 2012 and 2014 outbreaks computed by the cgMLST analysis confirmed that the MDR *A. baumannii* outbreaks in the caICU were two independent events. These findings suggested that the cgMLST study and SNP phylogeny provided the optimal resolution in differentiation outbreak strains. Similar to our findings, the cgMLST analysis of nosocomial infections associated with carbapenem-resistant *A. baumannii* from an Italian ICU was able to show two clonal clusters, whereas their traditional typing results suggested one cluster [30]. The authors also agreed that the cgMLST is a valuable tool that provides the highest discriminatory power in studying clonal relations among outbreak strains. From our study, both SNP-based results with filtered recombination and cgMLST analyses are compatible in outbreak investigations. In the case of the SNP-based analysis, it required an additional step to filter the recombination that can affect the conclusion of the outbreak investigation. Thus, cgMLST covers the limitation of an SNP-based approach by reducing the effect of recombination. It can be beneficial to cooperate with the cgMLST scheme in the WGS-based routine surveillance since the software used for cgMLST is user-friendly and does not require in-depth bioinformatics skills to compute the analysis. These findings indicate that the interpretation based on conventional MLST and phenotypic resistance profiles are insufficient to study the epidemiology and transmission chain of a limited number of *A. baumannii* infections in a veterinary healthcare setting.

### 3.2. Genetic Differences between Clinical and Environmental A. baumanni Isolates 

The comparative genome analysis of outbreak isolates and reference genomes demonstrated the differences in gene content, including antimicrobial resistance genes, virulence factors, and mobile genetic elements. We identified biofilm-associated virulent genes such as *bap* and *bauA* only in ST2 isolates. In addition, the dog genome obtained from the wound infection from 2012 carried a virulence gene (*traT*), which encoded the R6-5 plasmid-specified outer membrane protein that was demonstrated to mediate serum resistance in bloodstream infections [31]. This gene is not universally present [32], but a recent study showed *traT* was found in 80% of carbapenemase-producing *A. baumannii* isolates in Iran [33]. The differences in gene content among clinical isolates might be due to phage insertion or deletion since we observed variation in phage components among outbreak isolates. Our findings cannot explain the mechanisms in which these genetic differences impact the pathogenesis in dog patients.

Although the MDR phenotypes were identical between the 2012 and 2014 outbreak isolates, only the *catA1* gene encoding for chloramphenicol resistance was present in the 2014 isolates. However, in *A. baumannii,* the *catA1* gene is redundant, as all isolates are intrinsically resistant to chloramphenicol due to the CraA efflux pump [34]. All isolates carried resistance-nodulation-division (RND) efflux pumps (AdeABC) that previously showed that over-expression of efflux pumps had significant effect on susceptibility to some antimicrobials including beta-lactams, fluoroquinolones, and aminoglycosides [35]. However, a recent study showed that efflux-pump overexpression played a less significant role in the development of carbapenem resistance in *A. baumannii*, whereas biofilm production was strongly associated with carbapenem resistance phenotype [36]. In the current study, all MDR ST2 isolates carried biofilm-associated protein (*bap*), and there was no molecular detection of carbapenem resistance genes.

To explore the potential of *A. baumannii* as a zoonotic pathogen, we tried to identify differences in gene contents based on the host (i.e., human, dog, and environment). We could not identify host-specific genes. Our results implicate that *A. baumannii* may freely transmit between the animal and human host and cause infection without the requirement of host-specific factors. Host-specific genes may exist, and we might not have detected them with the limited number of isolates included in this study. From this study, we urge to include more isolates of animal origin in future research to carefully investigate the human-animal interplay of *A. baumannii*.

Environmental sampling from both years added additional value to the outbreak investigation. In 2012, *A. baumannii* was not traceable from the environment in the ICU, whereas it was recovered from surfaces in the ICU, neighboring rooms, and fur from a hospitalized dog in 2014. One surface isolate from the ICU treatment table was genetically identical to the 2014 patient isolates and belonged to the same clonal cluster (C2). This demonstrates the risk of environmental contamination and highlights the pre-existing challenge in eradicating *A. baumannii* from surfaces. Other environmental isolates displayed different sequence types without other ARGs besides beta-lactamase genes and mobile genetic elements except in a sample derived from the clinic operating room in the 2014 outbreak. This isolate belonged to ST239 carrying the *bla*OXA-51 carbapenemase gene and the putative plasmid harboring genes for type B chloramphenicol acetyltransferase. [37]. This putative plasmid type has never been reported in *Acinetobacter* species before. This plasmid-mediated chloramphenicol resistance mechanism is different from the 2014 outbreak isolates in which *catA1* is chromosomally located and encoded type A chloramphenicol acetyltransferase. The exchange of such plasmids between humans and companion animals is still unknown. ST239 was reported before in pets from France and a child from Tanzania; however, just as in our study, only single isolates of ST239 were described [38,39]. The detection of carbapenemase genes and putative plasmids encoding for drug resistance in the healthcare environment in this study is noteworthy. In addition, we identified the putative plasmids only in two environmental isolates (UKG-Inl-T1-1N-1, UKG-Inl-T1-4N-2) from 2014. All patient isolates carried mobile elements carrying aminoglycosides resistance genes *(aac(3)-Ia*, *ant(3″*), sulfonamide resistance gene (*sul1*), and *bla*TEM, suggesting the horizontal gene transfer of ARGs.

### 3.3. Methodological Considerations

The plasmid analysis in this study remained limited, given that the WGS was based on short paired-end sequencing. A combination of short contigs assembly with long-read sequencing can precisely determine whether the genes identified are encoded by chromosome or plasmid. In addition, we did not perform phenotypic analysis such as broth dilution and carbapenem inactivation methods to detect carbapenemase production of the isolates in this study [40]. However, whole-genome sequencing analysis revealed that there were no carbapenemase genes in patient genomes from both outbreaks. Another limitation was the interpretation of drug resistance in clinical isolates. Currently, there are no established veterinary-specific clinical breakpoints for Acinetobacter species and the standardized definitions for multi drug resistance are not widely available [41]. Thus, we used the general definition of multidrug resistance that has been widely used to characterize MDR in animal isolates [42] as opposed to the more comprehensive guideline developed for human medicine [43]. Thus, there is a need to develop a definition of drug resistance in veterinary medicine that can be used universally. Further work is required to study the diversity and abundance of *A. baumannii* in animal species and the horizontal transfer dynamics of virulence and AMR plasmids between the pathogenic and commensal strains.

### 3.4. The Importance of WGS-Based Surveillance in Animals and the Environment

In veterinary medicine, *A. baumannii* remains a neglected pathogen with limited data from strains originating from animals and their environment. Our study demonstrated that animals and humans share identical clones (ST2) and the same B-lactamase (*bla*OXA-66). Our study underlines the importance of genomic investigation combined with molecular clock determination in studying *A. baumannii* from animal origins. This easy-to-use and relatively cheap WGS and cgMLST platform could benefit WGS-based routine typing in outbreak management and surveillance in hospital settings. The implications from this study can increase awareness and help reduce transmission of MDR *A. baumannii* infections in small animal veterinary clinics.

## 4. Materials and Methods

### 4.1. Selection of Strains

A total of 11 *Acinetobacter baumannii* strains were isolated from dogs from the outbreaks in 2012 (*n* = 4), 2014 (*n* = 3), and environmental samples (*n* = 4) in 2014 in the calCU at the faculty of Veterinary Medicine of Utrecht University. Three reference strains belonging to European clones EC-I, EC- II, and EC-III were also sequenced and included in this study for comparison with study isolates for outbreak investigation.

### 4.2. Antimicrobial Susceptibility Testing (AST)

Antimicrobial susceptibility tests (ASTs) of the isolates were performed to determine the minimum inhibition concentration (MIC), using the microbroth dilution assay MICRONAUT-S (Merlin Diagnostika Gmbh, Bornheim, Germany). AST was performed as recommended by the manufacturer for inoculum preparation, broth composition, and incubation conditions. Customized MIC plates were used and read both visually and with a microplate reader (ThermoFisher scientific multiskan^TM^ FC Microplate Photometer, Hayward, CA, USA) using Thermo Scientific^TM^ SkanIt^TM^ software. The customized MIC plates include concentration ranges of the following antimicrobials: amoxicillin/clavulanic acid (AMC), ampicillin (AMP), cefepime (CEP), ceftiofur (CET), clindamycin (CLI), chloramphenicol (CMP), colistin (COL), cefoxitin (COX), cephalothin (CTN), enrofloxacin (ENR), erythromycin (ERY), fusidic acid (FUS), gentamicin (GEN), kanamycin (KAN), metronidazole (MTR), neomycin (NEO), nitrofurantoin (NFT), oxacillin (OXA), penicillin G (PEN), rifampicin (RAM), trimethoprim/sulfamethoxazole (T/S), and tetracycline (TET). AST results were interpreted according to Clinical Laboratory Standards Institute (CLSI) guidelines [44,45]. The multidrug resistance (MDR) was classified if the isolate was resistant to 3 or more antimicrobial classes [42] while isolates were defined as third-generation cephalosporins (3GC) resistant if resistance was found for third-generation cephalosporins (Ceftiofur, CET).

### 4.3. DNA Isolation and Quantification

DNA isolation was performed using the DNeasy^®^ UtraClean^®^ Microbial kit (Qiagen Gmbh, Germany) according to the manufacturer’s protocol. A total of 50 µL of DNA concentration was collected after DNA isolation and stored at 4 °C. A total of 1 µL of DNA from the sample with 199 µL of Qubit^®^ working solution from Qubit™ dsDNA HS Assay Kit (Invitrogen, Thermo Fisher Scientific, Waltham, MA, USA) was mixed to measure the concentration of DNA using Invitrogen Qubit Fluorometers (Thermo Fisher Scientific, Waltham, MA, USA).

### 4.4. Whole-Genome Sequencing and Genome Analysis

The sequencing of the *A. baumannii* isolates was performed using Illumina Miseq sequencing using 2 × 250 bp reads and 300 bp insert size by the Utrecht Sequencing Facility (USEQ, Utrecht, The Netherlands). The Illumina library was prepared with the final DNA concentration of 2 ng/µL using the Nextera XT Library Prep Kit (Illumina, San Diego, CA, USA) according to the manufacturer’s protocol.

The sequence data were trimmed with Trimmomatic v0.39 [46], assembled using SPAdes v3.14.1 [47] and annotated with Prokka v1.11 [48]. The quality of all sequences was checked with Checkm v1.1.3 [49], and only genomes with a contamination threshold of <5% and completeness threshold of >98% were included in the analysis. The comparative genome analysis was performed using Roary v3.13.0 [50], and a phylogenetic tree was constructed based on a core gene super alignment provided by Roary and single nucleotide polymorphism (SNP) detection using parsnp v1.2 [51]. Interactive Tree of Life (iTOL) v6.0 [52] was utilized to visualize the metadata of the genomes in a mid-rooted phylogenetic tree. Pan-genome data were visualized using the Phandango interactive tool [53]. ResFinder v4.0, the Comprehensive Antibiotic Resistance Database v3.1.4 [54] along with Mobile Element Finder v1.0.3 [55] (accessed on 9 December 2021), was used to identify the mobile genetic elements associated with antimicrobial resistance genes (ARGs). RFplasmid v0.0.16 [56] was used to estimate if the assembled contigs are plasmid or chromosomal, and any contigs with plasmid voting score > 0.6 were considered plasmid contigs. PlasmidFinder v2.0.1 [57] was used to identify the type of replicons.

### 4.5. Multi-Locus Sequence Typing

The sequence types (STs) of the genomes were assigned according to the Pasteur multi-locus sequence typing (MLST) schemes as previously described [28]. Subsequently, the core genome MLST (cgMLST) was performed using the cgMLST scheme [58] cooperated in the Ridom SeqSphere^+^ v8.0.2 software (Ridom GmBH, Münster, Germany) accessed on 1 October 2021. The clonal relationship of outbreak strains was visualized by a minimum spanning tree based on 2390 target alleles by the cgMLST scheme (paired-wise ignored missing values). The clonal cluster (CC) was defined based on the cgMLST scheme [58] where isolates sharing ≤10 different alleles in target genes were considered highly related (CC).

### 4.6. Time-Resolved Phylogeny Reconstruction

Relevant *A. baumannii* genomes with known isolation dates (*n* = 159) were obtained from Genbank (Supplementary Table S2). Among them, 6 genomes related to the outbreak isolates were selected from a phylogenetic tree based on SNP detection of the downloaded genomes (Supplementary Figure S2). This analysis included these additional ST-2 genomes (*n* = 6) from relevant literature [59,60,61,62,63,64] dated between 1982, when the EC-1 reference strain was isolated, and 2012 (Supplementary Table S2). Firstly, Gubbins v1.4.5 [65] predicted the recombination events in core genome alignment. Subsequently, the recombination regions were filtered, and the resulting super alignment of the 3144 genes without recombination signature was used in BEAST v1.8.4 [66] with the isolates dates as tip dates. BEAST was used to estimate the divergence dates using the BEAST XML generated by BEAUti [67]. The analysis was based on the generalized time-reversible (GTR) model without rate variation between sites and gamma correction as distance model, a Bayesian Skyline plot with 4 groups as demographic models, and a strict clock model. BEAST was run for 10,000,000 iterations with sampling. Tracer was used to evaluate the Effective Sample Sizes (ESS). ESS values > 200 were obtained.

## Figures and Tables

**Table 1:** Summary of epidemiological data and genomic characteristics of six clinical isolates from dogs and five environmental isolates from caICU.

Isolate	Source	Outbreak	Date	MLST [28]	AST *	Genome Coverage	Contigs	Genome Size (bp)
212092102901	dog 1-respiratory tract	2012	5 July 2012	ST2	MDR	92x	152	3,894,627
212062205001	dog 2-urinary tract	2012	22 June 2012	ST2	MDR	145x	154	3,902,040
212082004201	dog 3-urinary tract	2012	20 August 2012	ST2	MDR	144x	171	3,876,024
212090506901	dog 4-wound	2012	5 September 2012	ST2	MDR	148x	321	3,962,219
214030705701	dog 5-respiratory tract	2014	7 March 2014	ST2	MDR	97x	199	3,939,663
214031705301	dog 6-wound	2014	17 March 2014	ST2	MDR	104x	362	3,912,898
214032504901	ICU treatment table	2014	25 March 2014	ST2	MDR	162x	169	3,823,448
214032504501	medium care cage 9	2014	25 March 2014	-	3GC	144x	87	4,004,712
MCD-Ch-OK3-2PV-1	dog 7-commensal skin carriage	2014	25 March 2014	ST241	3GC	131x	73	3,932,237
UKG-Inl-T1-1N-1	clinic, operating table	2014	25 March 2014	ST239	3GC	145x	324	3,889,664
UKG-Inl-T1-4N-2	preparation room	2014	25 March 2014	ST837	3GC	88x	294	4,018,358
RUH-875	human-European Clone-I (EC-I)	Reference	1984	ST1	NA	100x	164	4,140,463
RUH-134	human-European Clone-II (EC-II)	Reference	1982	ST2	NA	127x	140	3,877,789
LUH-5875	human-European Clone-III (EC-III)	Reference	1997	ST3	NA	58x	115	3,833,285

Three human reference strains belonging to European clones (EC-I, EC-II, and EC-III) were included. * MDR: resistant to aminoglycosides, cephalosporins, chloramphenicol, enrofloxacin, penicillin, tetracycline, trimethoprim/sulfamethoxazole. 3GC: resistant to third-generation cephalosporins—unknown; NA: not available.

## Data Availability

The sequence data were deposited in the European Nucleotide Archive (ENA) under study accession number PRJEB19153.

## Supplementary Materials

The following are available online at https://www.mdpi.com/article/10.3390/pathogens11020123/s1, Figure S1: the description of A. baumannii cases in a companion animal intensive care unit (caICU) in 2012 and 2014.; Figure S2: the phylogenetic tree of isolates with different sequence types (ST); Table S1: antimicrobial susceptibility test results of the outbreak isolates from 2012 and 2014; Table S2: relevant genomes with known isolation date from literature; Table S3: the recombination regions and the single nucleotide polymorphism (SNP) difference between outbreak isolates.

## References

[1] Munoz-Price L.S., Weinstein R.A. (2008). Acinetobacter Infection. New Engl. J. Med..

[2] Endimiani A., Hujer K.M., Hujer A.M., Bertschy I., Rossano A., Koch C., Gerber V., Francey T., Bonomo R.A., Perreten V. (2011). *Acinetobacter baumannii* Isolates from Pets and Horses in Switzerland: Molecular Characterization and Clinical Data. J. Antimicrob. Chemother..

[3] Francey T., Gaschen F., Nicolet J., Burnens A.P. (2000). The Role of *Acinetobacter baumannii* as a Nosocomial Pathogen for Dogs and Cats in an Intensive Care Unit. J. Vet. Intern. Med..

[4] Karageorgopoulos D.E., Falagas M.E. (2008). Current Control and Treatment of Multidrug-Resistant *Acinetobacter baumannii* Infections. Lancet Infect. Dis..

[5] Müller S., Janßen T., Wieler L. (2014). Multidrug Resistant *Acinetobacter baumannii* in Veterinary Medicine—Emergence of an Underestimated Pathogen?. Berl. Münch. Tierärztl. Wochenschr..

[6] Bergogne-Bérézin E., Towner K.J. (1996). *Acinetobacter* spp. as Nosocomial Pathogens: Microbiological, Clinical, and Epidemiological Features. Clin. Microbiol. Rev..

[7] Guerra B., Fischer J., Helmuth R. (2014). An Emerging Public Health Problem: Acquired Carbapenemase-Producing Microorganisms Are Present in Food-Producing Animals, Their Environment, Companion Animals and Wild Birds. Vet. Microbiol..

[8] Wareth G., Neubauer H., Sprague L.D. (2019). *Acinetobacter baumannii*—A Neglected Pathogen in Veterinary and Environmental Health in Germany. Vet. Res. Commun..

[9] Fournier P.-E., Vallenet D., Barbe V., Audic S., Ogata H., Poirel L., Richet H., Robert C., Mangenot S., Abergel C. (2006). Comparative Genomics of Multidrug Resistance in *Acinetobacter baumannii*. PLoS Genet..

[10] Wong D., Nielsen T.B., Bonomo R.A., Pantapalangkoor P., Luna B., Spellberg B. (2017). Clinical and Pathophysiological Overview of *Acinetobacter* Infections: A Century of Challenges. Clin. Microbiol. Rev..

[11] World Health Organization WHO Publishes List of Bacteria for Which New Antibiotics Are Urgently Needed. https://www.who.int/news/item/27-02-2017-who-publishes-list-of-bacteria-for-which-new-antibiotics-are-urgently-needed.

[12] Klotz P., Higgins P.G., Schaubmar A.R., Failing K., Leidner U., Seifert H., Scheufen S., Semmler T., Ewers C. (2019). Seasonal Occurrence and Carbapenem Susceptibility of Bovine *Acinetobacter baumannii* in Germany. Front. Microbiol..

[13] Kempf M., Abdissa A., Diatta G., Trape J.-F., Angelakis E., Mediannikov O., La Scola B., Raoult D. (2012). Detection of *Acinetobacter baumannii* in Human Head and Body Lice from Ethiopia and Identification of New Genotypes. Int. J. Infect. Dis..

[14] Mitchell K.E., Turton J.F., Lloyd D.H. (2018). Isolation and Identification of *Acinetobacter* spp. from Healthy Canine Skin. Vet. Dermatol..

[15] Jacobmeyer L., Stamm I., Semmler T., Ewers C. (2021). First Report of NDM-1 in an Acinetobacter Baumannii Strain from a Pet Animal in Europe. J. Glob. Antimicrob. Resist..

[16] Nocera F.P., Attili A.-R., De Martino L. (2021). Acinetobacter Baumannii: Its Clinical Significance in Human and Veterinary Medicine. Pathogens.

[17] Weinberg S.E., Villedieu A., Bagdasarian N., Karah N., Teare L., Elamin W.F. (2020). Control and Management of Multidrug Resistant *Acinetobacter baumannii*: A Review of the Evidence and Proposal of Novel Approaches. Infect. Prev. Pract..

[18] Van der Kolk J.H., Endimiani A., Graubner C., Gerber V., Perreten V. (2019). Acinetobacter in Veterinary Medicine, with an Emphasis on *Acinetobacter baumannii*. J. Glob. Antimicrob. Resist..

[19] Ewers C., Bethe A., Semmler T., Guenther S., Wieler L.H. (2012). Extended-Spectrum β-Lactamase-Producing and AmpC-Producing Escherichia Coli from Livestock and Companion Animals, and Their Putative Impact on Public Health: A Global Perspective. Clin. Microbiol. Infect..

[20] Davis M.F., Iverson S.A., Baron P., Vasse A., Silbergeld E.K., Lautenbach E., Morris D.O. (2012). Household Transmission of Meticillin-Resistant *Staphylococcus aureus* and Other Staphylococci. Lancet Infect. Dis..

[21] Zordan S. (2011). Multidrug-Resistant *Acinetobacter baumannii* in Veterinary Clinics, Germany. Emerg. Infect. Dis..

[22] Wu T.-L., Su L.-H., Leu H.-S., Chiu C.-H., Chiu Y.-P., Chia J.-H., Kuo A.-J., Sun C.-F. (2002). Molecular Epidemiology of Nosocomial Infection Associated with Multi-Resistant *Acinetobacter baumannii* by Infrequent-Restriction-Site PCR. J. Hosp. Infect..

[23] Fitzpatrick M.A., Ozer E.A., Hauser A.R. (2016). Utility of Whole-Genome Sequencing in Characterizing Acinetobacter Epidemiology and Analyzing Hospital Outbreaks. J. Clin. Microbiol..

[24] Rafei R., Osman M., Dabboussi F., Hamze M. (2019). Update on the Epidemiological Typing Methods for *Acinetobacter baumannii*. Future Microbiol..

[25] Lewis T., Loman N.J., Bingle L., Jumaa P., Weinstock G.M., Mortiboy D., Pallen M.J. (2010). High-Throughput Whole-Genome Sequencing to Dissect the Epidemiology of *Acinetobacter baumannii* Isolates from a Hospital Outbreak. J. Hosp. Infect..

[26] Köser C.U., Holden M.T.G., Ellington M.J., Cartwright E.J.P., Brown N.M., Ogilvy-Stuart A.L., Hsu L.Y., Chewapreecha C., Croucher N.J., Harris S.R. (2012). Rapid Whole-Genome Sequencing for Investigation of a Neonatal MRSA Outbreak. New Engl. J. Med..

[27] Snitkin E.S., Zelazny A.M., Thomas P.J., Stock F., Henderson D.K., Palmore T.N., Segre J.A., NISC Comparative Sequencing Program (2012). Tracking a Hospital Outbreak of Carbapenem-Resistant *Klebsiella pneumoniae* with Whole-Genome Sequencing. Sci. Transl. Med..

[28] Diancourt L., Passet V., Nemec A., Dijkshoorn L., Brisse S. (2010). The Population Structure of *Acinetobacter baumannii*: Expanding Multiresistant Clones from an Ancestral Susceptible Genetic Pool. PLoS ONE.

[29] Peleg A.Y., Seifert H., Paterson D.L. (2008). *Acinetobacter baumannii*: Emergence of a Successful Pathogen. Clin. Microbiol. Rev..

[30] Venditti C., Vulcano A., D’Arezzo S., Gruber C.E.M., Selleri M., Antonini M., Lanini S., Marani A., Puro V., Nisii C. (2019). Epidemiological Investigation of an *Acinetobacter baumannii* Outbreak Using Core Genome Multilocus Sequence Typing. J. Glob. Antimicrob. Resist..

[31] Montenegro M.A., Bitter-Suermann D., Timmis J.K., Aguero M.E., Cabello F.C., Sanyal S.C., Timmis K.N. (1985). TraT Gene Sequences, Serum Resistance and Pathogenicity-Related Factors in Clinical Isolates of *Escherichia coli* and Other Gram-Negative Bacteria. Microbiology.

[32] Liu C., Chang Y., Xu Y., Luo Y., Wu L., Mei Z., Li S., Wang R., Jia X. (2018). Distribution of Virulence-Associated Genes and Antimicrobial Susceptibility in Clinical *Acinetobacter baumannii* Isolates. Oncotarget.

[33] Mohajeri P., Sharbati S., Farahani A., Rezaei Z. (2016). Evaluate the Frequency Distribution of Nonadhesive Virulence Factors in Carbapenemase-Producing *Acinetobacter baumannii* Isolated from Clinical Samples in Kermanshah. J. Nat. Sci. Biol. Med..

[34] Roca I., Marti S., Espinal P., Martínez P., Gibert I., Vila J. (2009). CraA, a Major Facilitator Superfamily Efflux Pump Associated with Chloramphenicol Resistance in *Acinetobacter baumannii*. Antimicrob. Agents Chemother..

[35] Donadu M.G., Zanetti S., Nagy Á.L., Barrak I., Gajdács M. (2021). Insights on Carbapenem-Resistant *Acinetobacter baumannii*: Phenotypic Characterization of Relevant Isolates. Acta Biol. Szeged..

[36] Donadu M.G., Mazzarello V., Cappuccinelli P., Zanetti S., Madléna M., Nagy Á.L., Stájer A., Burián K., Gajdács M. (2021). Relationship between the Biofilm-Forming Capacity and Antimicrobial Resistance in Clinical *Acinetobacter baumannii* Isolates: Results from a Laboratory-Based In Vitro Study. Microorganisms.

[37] Schwarz S., Kehrenberg C., Doublet B., Cloeckaert A. (2004). Molecular Basis of Bacterial Resistance to Chloramphenicol and Florfenicol. FEMS Microbiol. Rev..

[38] Belmonte O., Pailhoriès H., Kempf M., Gaultier M.P., Lemarié C., Ramont C., Joly-Guillou M.L., Eveillard M. (2014). High Prevalence of Closely-Related *Acinetobacter baumannii* in Pets According to a Multicentre Study in Veterinary Clinics, Reunion Island. Vet. Microbiol..

[39] Moyo S.J., Manyahi J., Hubbard A.T.M., Byrne R.L., Masoud N.S., Aboud S., Manji K., Blomberg B., Langeland N., Roberts A.P. (2021). Molecular Characterisation of the First New Delhi Metallo-β-Lactamase 1-Producing *Acinetobacter baumannii* from Tanzania. Trans. R. Soc. Trop. Med. Hyg..

[40] Uechi K., Tada T., Shimada K., Kuwahara-Arai K., Arakaki M., Tome T., Nakasone I., Maeda S., Kirikae T., Fujita J. (2017). A Modified Carbapenem Inactivation Method, CIMTris, for Carbapenemase Production in Acinetobacter and Pseudomonas Species. J. Clin. Microbiol..

[41] Sweeney M.T., Lubbers B.V., Schwarz S., Watts J.L. (2018). Applying Definitions for Multidrug Resistance, Extensive Drug Resistance and Pandrug Resistance to Clinically Significant Livestock and Companion Animal Bacterial Pathogens. J. Antimicrob. Chemother..

[42] Schwarz S., Silley P., Simjee S., Woodford N., van Duijkeren E., Johnson A.P., Gaastra W. (2010). Assessing the Antimicrobial Susceptibility of Bacteria Obtained from Animals. Vet. Microbiol..

[43] Magiorakos A.-P., Srinivasan A., Carey R.B., Carmeli Y., Falagas M.E., Giske C.G., Harbarth S., Hindler J.F., Kahlmeter G., Olsson-Liljequist B. (2012). Multidrug-Resistant, Extensively Drug-Resistant and Pandrug-Resistant Bacteria: An International Expert Proposal for Interim Standard Definitions for Acquired Resistance. Clin. Microbiol. Infect..

[44] CLSI (2013). Performance Standards for Antimicrobial Disk and Dilution Susceptibility Tests for Bacteria Isolated from Animals.

[45] CLSI (2014). Performance Standards for Antimicrobial Susceptibility Testing.

[46] Bolger A.M., Lohse M., Usadel B. (2014). Trimmomatic: A Flexible Trimmer for Illumina Sequence Data. Bioinformatics.

[47] Bankevich A., Nurk S., Antipov D., Gurevich A.A., Dvorkin M., Kulikov A.S., Lesin V.M., Nikolenko S.I., Pham S., Prjibelski A.D. (2012). SPAdes: A New Genome Assembly Algorithm and Its Applications to Single-Cell Sequencing. J. Comput. Biol..

[48] Seemann T. (2014). Prokka: Rapid Prokaryotic Genome Annotation. Bioinformatics.

[49] Parks D.H., Imelfort M., Skennerton C.T., Hugenholtz P., Tyson G.W. (2015). CheckM: Assessing the Quality of Microbial Genomes Recovered from Isolates, Single Cells, and Metagenomes. Genome Res..

[50] Page A.J., Cummins C.A., Hunt M., Wong V.K., Reuter S., Holden M.T.G., Fookes M., Falush D., Keane J.A., Parkhill J. (2015). Roary: Rapid Large-Scale Prokaryote Pan Genome Analysis. Bioinformatics.

[51] Treangen T.J., Ondov B.D., Koren S., Phillippy A.M. (2014). The Harvest Suite for Rapid Core-Genome Alignment and Visualization of Thousands of Intraspecific Microbial Genomes. Genome Biol..

[52] Letunic I., Bork P. (2016). Interactive Tree of Life (ITOL) v3: An Online Tool for the Display and Annotation of Phylogenetic and Other Trees. Nucleic Acids Res..

[53] Hadfield J., Croucher N.J., Goater R.J., Abudahab K., Aanensen D.M., Harris S.R. (2018). Phandango: An Interactive Viewer for Bacterial Population Genomics. Bioinformatics.

[54] Alcock B.P., Raphenya A.R., Lau T.T.Y., Tsang K.K., Bouchard M., Edalatmand A., Huynh W., Nguyen A.-L.V., Cheng A.A., Liu S. (2019). CARD 2020: Antibiotic Resistome Surveillance with the Comprehensive Antibiotic Resistance Database. Nucleic Acids Res..

[55] Johansson M.H.K., Bortolaia V., Tansirichaiya S., Aarestrup F.M., Roberts A.P., Petersen T.N. (2021). Detection of Mobile Genetic Elements Associated with Antibiotic Resistance in *Salmonella enterica* Using a Newly Developed Web Tool: MobileElementFinder. J. Antimicrob. Chemother..

[56] Van der Graaf-van Bloois L., Wagenaar J.A., Zomer A.L. (2021). RFPlasmid: Predicting Plasmid Sequences from Short-Read Assembly Data Using Machine Learning. Microb. Genom..

[57] Thomsen M.C.F., Ahrenfeldt J., Cisneros J.L.B., Jurtz V., Larsen M.V., Hasman H., Aarestrup F.M., Lund O. (2016). A Bacterial Analysis Platform: An Integrated System for Analysing Bacterial Whole Genome Sequencing Data for Clinical Diagnostics and Surveillance. PLoS ONE.

[58] Higgins P.G., Prior K., Harmsen D., Seifert H. (2017). Development and Evaluation of a Core Genome Multilocus Typing Scheme for Whole-Genome Sequence-Based Typing of *Acinetobacter baumannii*. PLoS ONE.

[59] Sahl J.W., Gillece J.D., Schupp J.M., Waddell V.G., Driebe E.M., Engelthaler D.M., Keim P. (2013). Evolution of a Pathogen: A Comparative Genomics Analysis Identifies a Genetic Pathway to Pathogenesis in Acinetobacter. PLoS ONE.

[60] Snitkin E.S., Zelazny A.M., Montero C.I., Stock F., Mijares L., Murray P.R., Segre J.A., Mullikin J., Blakesley R., NISC Comparative Sequence Program (2011). Genome-Wide Recombination Drives Diversification of Epidemic Strains of *Acinetobacter baumannii*. Proc. Natl. Acad. Sci. USA.

[61] Zarrilli R., Giannouli M., Rocco F., Loman N.J., Haines A.S., Constantinidou C., Pallen M.J., Triassi M., Di Nocera P.P. (2011). Genome Sequences of Three *Acinetobacter baumannii* Strains Assigned to the Multilocus Sequence Typing Genotypes ST2, ST25, and ST78. J. Bacteriol..

[62] Adams M.D., Goglin K., Molyneaux N., Hujer K.M., Lavender H., Jamison J.J., MacDonald I.J., Martin K.M., Russo T., Campagnari A.A. (2008). Comparative Genome Sequence Analysis of Multidrug-Resistant *Acinetobacter baumannii*. J. Bacteriol..

[63] Chan A.P., Sutton G., DePew J., Krishnakumar R., Choi Y., Huang X.-Z., Beck E., Harkins D.M., Kim M., Lesho E.P. (2015). A Novel Method of Consensus Pan-Chromosome Assembly and Large-Scale Comparative Analysis Reveal the Highly Flexible Pan-Genome of *Acinetobacter baumannii*. Genome Biol..

[64] Zurawski D.V., Thompson M.G., McQueary C.N., Matalka M.N., Sahl J.W., Craft D.W., Rasko D.A. (2012). Genome Sequences of Four Divergent Multidrug-Resistant *Acinetobacter baumannii* Strains Isolated from Patients with Sepsis or Osteomyelitis. J. Bacteriol..

[65] Croucher N.J., Page A.J., Connor T.R., Delaney A.J., Keane J.A., Bentley S.D., Parkhill J., Harris S.R. (2015). Rapid Phylogenetic Analysis of Large Samples of Recombinant Bacterial Whole Genome Sequences Using Gubbins. Nucleic Acids Res..

[66] Drummond A.J., Rambaut A. (2007). BEAST: Bayesian Evolutionary Analysis by Sampling Trees. BMC Evol. Biol..

[67] Drummond A.J., Suchard M.A., Xie D., Rambaut A. (2012). Bayesian Phylogenetics with BEAUti and the BEAST 1.7. Mol. Biol. Evol..

