# Supplementary material for: Genomic Investigation of Two Acinetobacter baumannii Outbreaks in a Veterinary Intensive Care Unit in The Netherlands
Source: Pathogens. 2022 Jan 20;11(2):123. doi: 10.3390/pathogens11020123 (PMC8875366; doi:10.3390/pathogens11020123)
Supplement: Supplementary file 1 [file pathogens-11-00123-s001.zip › Figure S1 and S2.pdf]

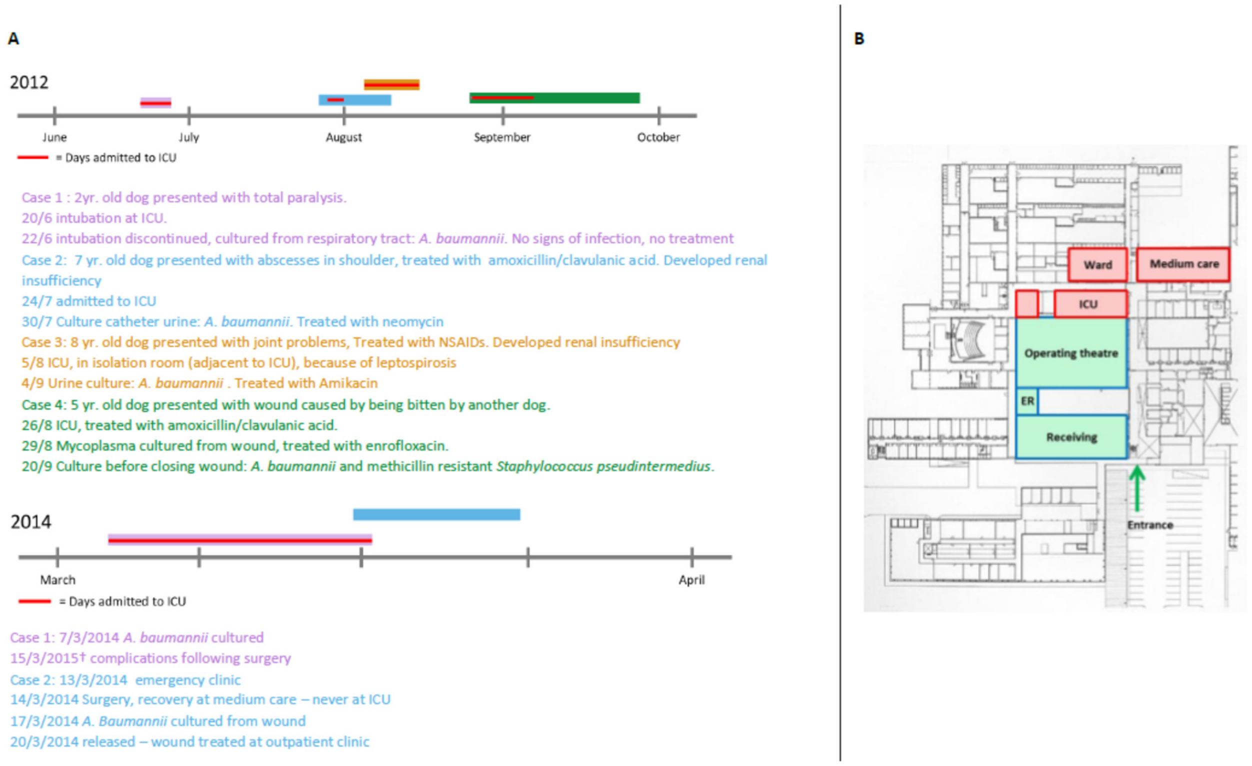

Figure S1. The description of *A. baumannii* cases in companion animal intensive care unit (caICU) in 2012 and 2014. (**A**) The schematic overview of patients infected with *A. baumannii* in caICU 2012 and 2014. (**B**) The floorplan of companion animal intensive care unit (caICU) at the Utrecht University, the Netherlands.

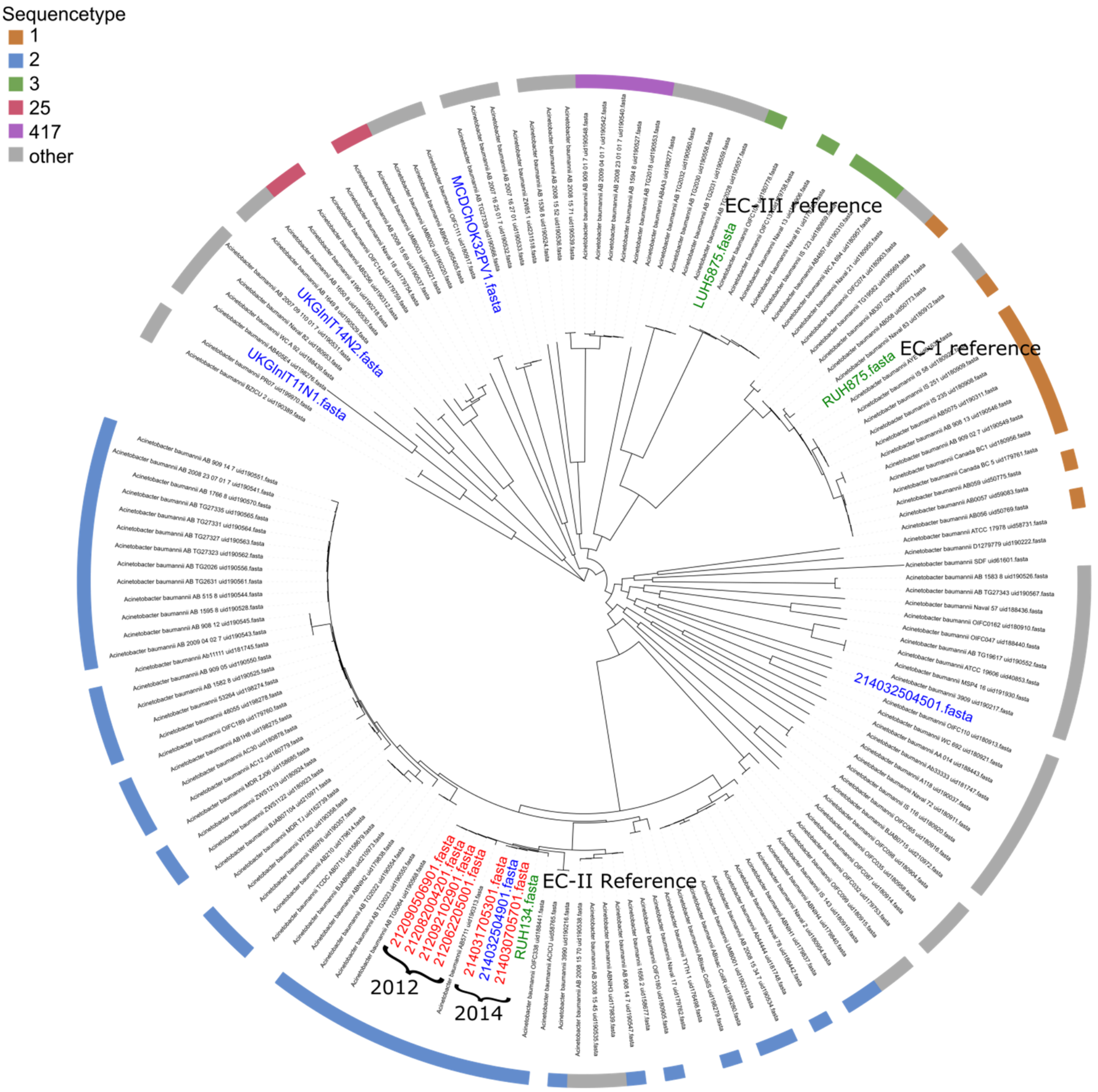

Figure S2. Phylogenetic tree of isolates sequenced in this study and isolates observed in literature. Different label colors represented different sources of samples (clinical isolates in red, environmental isolates in blue, and the EC-I, EC-II and EC-III reference isolates in green).
